# Accessible, Equitable, and Personalized Care for Autistic Individuals

**DOI:** 10.3390/jcm11175217

**Published:** 2022-09-03

**Authors:** Karen Bearss, Soo-Jeong Kim, Jill Locke

**Affiliations:** 1 Psychiatry and Behavioral Sciences, University of Washington, Seattle, WA 98195, USA; 2 Seattle Children’s Autism Center, Seattle Children’s Hospital, Seattle, WA 98105, USA

## 1. Access to Healthcare Services

When it comes to service accessibility for autistic individuals, there exists a pipeline problem. This starts with the assumption that an individual can even receive a diagnosis of autism spectrum disorder (ASD), acknowledging it can take months or even years for this to occur. For example, in the United States, the average age of diagnosis is 4 years of age, despite the research that supports the ability of clinicians to reliably diagnose autism as early as 24 months [1], and noting that this average age of diagnosis is even higher for underserved populations [2].

Once an individual receives a diagnosis of ASD, the clinician typically provides a range of targeted, evidence-based treatment recommendations for the autistic individual and their family members. Herein lies the pipeline problem. While clinicians may emphasize “the more hours of therapy the better” and “time is of the essence, the sooner you can get into therapy better,” families leave diagnostic visits and run into a metaphoric brick wall as they attempt to access the very services that the clinician has adamantly recommended. This brick wall, in reality, is a series of barriers, including, but not limited to the following: (1) extended wait times as the result of high service demand and too few qualified providers; (2) high costs of treatment related to the recommended service intensity (upwards of 15 to 25 h/week); (3) lack of transportation and/or distance to travel, noting that many autism services are located in specialized centers in urban hubs; (4) access to services that are only available during typical work hours; and/or (5) lack of access to trained specialists in one’s local community. Thus, a primary need for autistic individuals and their families is the expansion of evidence-based interventions that are also time-limited, cost-effective, widely available, and easy to access.

## 2. Equitable, Personalized Care for Autistic Individuals

This call for expanded access to services runs in parallel to a related call to promote a personalized care approach to treatment for autistic individuals, which was highlighted in a recent seminal article published in *The Lancet* [3]. In the service model roadmap presented in the article, healthcare providers are recommended to start a course of care by first having the caregivers and autistic individual each identify which priority diagnoses and conditions require services. Treatment needs are selected, and goals are defined, with incorporation of the factors that may influence the likelihood of treatment success (e.g., safety issues, life events, developmental needs of the autistic individual, preference for medical vs. behavioral strategies). Consideration is given to both individual and family factors that might inform care, such as treatment modality preferences (e.g., in person, telehealth, group-based), service intensity needs (e.g., outpatient, inpatient) and access points of care (e.g., school, home, community clinic), noting that the provider should work to reconcile the challenges around accessing a particular service care model or when there are differences in caregiver vs. autistic individual preferences around care plan approaches. 

The goal is to determine a model of care that creates alignment and fits between the individual and family needs, preferences, and priorities. In other words, we must find a balance between a family’s priority for care and the resources available in their community, and tailor the care we provide to meet the needs of those we serve in a truly individualized way. To accomplish this, we would argue that truly responsive service care provisions for autistic individuals would advance the intersect between opportunities for highly personalized care and the ability to improve models of care that promote accessibility in the community. This should be on the forefront when developing care packages for individuals and caregivers. There are several new clinical and research efforts that are underway and are designed to expand options for accessible care for autistic individuals and their families that also increase the ability to move toward a personalized care model. 

## 3. Group Intervention Models

A long-standing intervention modality that has the benefit of increasing access to care is treatment delivery in a group format. In the context of challenges to accessing care, group formats allow for the provision of care to a greater number of individuals at once (addressing high service demand), allowing providers to reach more individuals more quickly, potentially reducing waitlist times and costs of treatment, noting that provider costs may be shared across group participants. 

In tying together group treatment modalities to the goal of personalized care models, it is important to recognize that there may be individuals who prefer participating a in group treatment modality over individual treatment, as groups provide the individual with a unique opportunity to connect to a built-in community network of support as part of their care. For autistic individuals, social skills training is a common intervention recommendation, which is best addressed when provided with opportunities to interact with other peers as a means to practice targeted skills. In fact, several evidence-based interventions for autistic youth involve group formats, including the PEERS program [4] and Facing Your Fears [5].

When considering the needs of caregivers of autistic individuals, there is ample research that describes the common feelings of social isolation [6]. The opportunity for caregivers to connect with other families experiencing the unique needs of raising an autistic child can dramatically reduce that sense of isolation [7]. For example, a caregiver that participates in a group intervention for autistic youth with challenging behaviors may describe a really challenging moment from the week prior and have other caregivers in the group nod their heads as a way to validate and acknowledge the situation as if to say, “That was me last week. I understand that experience”. There is an opportunity for building a community and support in the context of group modalities. 

## 4. Telehealth

Telehealth (also known as ‘telepractice’ or ‘telemedicine’) uses communication technologies (e.g., computer-based videoconferencing and the internet) that allow specialists to consult or deliver services in real-time over a geographical distance [8]. While delivery of care via telehealth has been available for decades, it remained a relatively novel modality for autism treatment, until COVID-19 related restrictions required a sudden and expansive shift in care delivery. 

The benefit of telehealth is it may directly target access concerns, including lack of transportation, long travel distances to specialized autism centers and/or lack of access to trained specialists in one’s local community. Another important benefit of telehealth involves the improved ability to include the autistic individual, as well as parents and other caregivers (e.g., grandparents, siblings) and treatment providers (therapists) in service provisions. This runs counter to the challenges related to in-person care models, where it can be difficult to involve more than one or two individuals (e.g., parent and child; two caregivers) to join a treatment session, as other individuals may have responsibilities that interfere with their ability to participate (e.g., at work, caring for other children at home). Now with telehealth, one caregiver can join an appointment from home, another can call in from work, etc., providing attendance flexibility and increased opportunity for broader family systems and other providers (e.g., educators) to engage in treatment. 

Telehealth also opens opportunities for various care providers to collaborate and coordinate care across service systems. This is important, as caregivers of autistic youth typically are required to juggle multiple appointments across fragmented systems of care, noting that an autistic individual undergoes an average of four to seven treatments at any one time, with greater symptom severity resulting in even greater service use [9,10]. 

Despite the benefits of telehealth service delivery models, it is important to acknowledge that telehealth may not be the best platform for care for all individuals or clinical teams, for example families with limited access to technology. In line with the personalized care approach, the idea is that the autistic individual and their family are presented with the choice to engage in treatment through this modality.

## 5. Digital Technologies

An emerging phenomenon is the use of interactive digital technologies to enhance access to care for autistic individuals and their families. This approach allows for service seekers to reduce, or altogether bypass, the need for direct care provision through providers by instead gaining direct access to evidence-based tools and therapies, eliminating potential wait times and travel requirements, often at reduced costs, and access to care at times of convenience (e.g., after work hours). For example, the University of California, Davis developed the Autism Distance Education Parent Training (ADEPT) program (https://health.ucdavis.edu/mindinstitute/centers/cedd/adept.html, accessed on 22 August 2022), which allows caregivers to engage in up to 10 interactive, self-paced, online learning modules, which provide tools and training to more effectively teach their autistic child functional skills, using applied behavior analysis (ABA) techniques. ADEPT provides the advantage of being accessible online at no cost and is available in multiple languages. 

Research on the use of digital technologies is only now emerging, and with mixed results; the most predominant finding suggests that self-guided programs alone may produce lower rates of treatment engagement and completion when compared to programs where therapist engagement is provided [11,12]. Thus, there are continued questions regarding the ways to improve the efficacy of and engagement with digital technologies. With the growing number of technology-based programs under development, their ability to create unique opportunities for autistic individuals and their families to engage in treatment at their convenience, thus aligning with the principle of personalized access to care, should continue to be explored. 

## 6. Summary

Autism spectrum disorder is highly heterogeneous and requires personalized, evidence-based assessments and interventions that can improve the outcomes of autistic individuals and their families [3]. Autism intervention research is still in its relative infancy, with the majority of large-scale trials published within the past 10 to 15 years [13,14]. Questions remain regarding the replicability of clinical outcomes of newly established evidence-based interventions across novel modalities (group format, telehealth delivery, digital apps and self-guided trainings). These questions are worth exploring, as the gain with these varied intervention modalities, aside from increased accessibility, allow for increased flexibility to approach care planning in alignment with the personalized care model. This is a truly exciting time to observe the broadening clinical and research efforts that are designed to address the longstanding challenges around ensuring autistic individuals and their families are connected to the very care that they need and more importantly, the care that they deserve.
